# The ABCB Multidrug Resistance Proteins Do Not Contribute to Ivermectin Detoxification in the Colorado Potato Beetle, *Leptinotarsa decemlineata* (Say)

**DOI:** 10.3390/insects11020135

**Published:** 2020-02-20

**Authors:** Grant Favell, Jeremy N. McNeil, Cam Donly

**Affiliations:** 1 Department of Biology, University of Western Ontario, London, ON N6A 3K7, Canada; 2 London Research and Development Centre, Agriculture and Agri-Food Canada, London, ON N5V 4T3, Canada

**Keywords:** ATP-binding cassette proteins, insecticide defense, RNAi

## Abstract

The Colorado potato beetle, *Leptinotarsa decemlineata* (Say), is a significant agricultural pest that has developed resistance to many insecticides that are used to control it. Investigating the mechanisms of insecticide detoxification in this pest is important for ensuring its continued control, since they may be contributors to such resistance. Multidrug resistance (MDR) genes that code for the ABCB transmembrane efflux transporters are one potential source of insecticide detoxification activity that have not been thoroughly examined in *L. decemlineata*. In this study, we annotated the ABCB genes found in the *L. decemlineata* genome and then characterized the expression profiles across midgut, nerve, and Malpighian tubule tissues of the three full transporters identified. To investigate if these genes are involved in defense against the macrocyclic lactone insecticide ivermectin in this insect, each gene was silenced using RNA interference or MDR protein activity was inhibited using a chemical inhibitor, verapamil, before challenging the insects with a dose of ivermectin. Survival of the insects did not significantly change due to gene silencing or protein inhibition, suggesting that MDR transporters do not significantly contribute to defense against ivermectin in *L. decemlineata*.

## 1. Introduction

The Colorado potato beetle, *Leptinotarsa decemlineata* (Say), is a major pest of solanaceous crops with a large geographical distribution that includes much of North America, and areas of Europe, and Asia, with potential to spread elsewhere [1,2]. Control of *L. decemlineata* often includes the use of chemical insecticides; however, due to extensive exposure to insecticides, this species has developed resistance to 55 different products in 13 different chemical groups [3]. Understanding the molecular basis of such resistance is an essential first step toward the development of new and sustainable strategies for the continued management of this pest.

One of many mechanisms by which insects respond to xenobiotic insult, including insecticide exposure, is through metabolic detoxification and subsequent excretion [4]. Enzymes such as esterases, glutathione-S-transferases (GSTs), UDP-glycosyltransferases (UGTs), and cytochrome P450-dependent monooxygenases (CYPs) metabolize harmful molecules to reduce their toxicity or make them more easily transported (phase I and II reactions), while transmembrane efflux transporters, such as multidrug resistance (MDR) proteins, facilitate their elimination or sequestration (phase III reactions). MDRs are members of the ATP-binding cassette (ABC) transporter superfamily, an ancient protein family consisting of transmembrane transporters that are present in archaea [5] bacteria [6] and eukaryotes [7]. They are defined by their ATP-binding cassette domain, which enables energy acquisition from ATP for active transport. MDRs are found within subfamily B of the ABC transporters (ABCB), known as the MDR/TAP subfamily, as it also includes ‘transporter associated with antigen processing’ (TAP) proteins. Genes for MDR proteins are typically expressed in barrier tissues such as digestive and blood-brain barrier tissues, as well as in tissues performing detoxification or regulatory functions [8,9,10,11,12,13,14,15]. In insects, these tissues include midgut, central nervous system (CNS), and Malpighian tubules (MT). MDR transporters have broad substrate specificity, so they are responsible for the efflux of a large variety of molecules, often including toxins.

MDR proteins have been intensively studied in humans because upregulation of MDR genes confers the multidrug resistance phenotype to cancer cells [16], but since they are highly conserved between organisms, their function is now being explored with respect to insect defenses against [17,18,19,20,21,22,23], and resistance to [24,25,26,27,28,29,30], insecticides. The development of insecticide resistance is frequently driven by amplified levels of natural defense processes, including those of both detoxification and excretion [31]. Thus, investigation of innate defense mechanisms present at lower levels in susceptible strains, which provide pre-adaptive defense capability in such insects, can yield insight into the process of resistance development.

ABC transporters, including MDRs, have been identified as resistance factors in insecticide resistant strains of tobacco budworm, *Heliothis virescens* [32], spider mite, *Tetranychus urticae* [33], small brown planthopper, *Laodelphax striatellus* [34], and the leaf beetle *Chrysomela tremula* [35], all of which are agricultural pests. Such resistance typically involves either inducible or constitutively increased expression of one or more transporter genes. A review by Dermauw and Van Leeuwen [25] reported that MDR transporters contributed to resistance to carbamates, macrocyclic lactones, organochlorines, organophosphates, pyrethroids, and Cry1A toxin in a diverse array of arthropod species. A variety of assays, including in vivo knockdown of gene expression and the use of pharmacological inhibitors of transporter activity, such as verapamil, were used to link these genes and proteins to such resistance. Although MDR transporter substrate specificity is broad, it can differ between different proteins and species. Macrocyclic lactones, such as ivermectin, can be useful indicators of MDR transporter contribution to insecticide tolerance because they have insecticidal activity and are MDR substrates in a wide variety of species from mammals [36] to nematodes [37]. Since it is a conserved substrate in many different species, ivermectin is a useful starting point for studying MDR activity in species whose MDR genes or proteins have not yet been investigated.

Despite the significance of *L. decemlineata* as an agricultural pest and its ability to develop resistance to a variety of insecticides, its MDR genes have not been closely investigated for any role in defense against insecticides. Using the recently published *L. decemlineata* genome [38], we identified the genes for three full transporters (among a total complement of seven ABCB family genes), and characterized their expression across a variety of tissues. The genes for these full transporters were then individually downregulated using double-stranded RNA (dsRNA)-mediated RNA interference (RNAi) or MDR proteins themselves were inhibited using the chemical inhibitor, verapamil, before challenging the insects with the insecticide ivermectin, to determine if MDR gene expression and protein activity contribute to defense against ivermectin in *L. decemlineata*.

## 2. Materials and Methods

### 2.1. Insect Rearing

All insects used came from laboratory colonies reared in the London Research and Development Centre (LRDC), Agriculture and Agri-Food Canada (AAFC) in London, Ontario, Canada. The insects were collected in 1991 from the LRDC research farm and reared for over 180 generations with no exposure to insecticides. Insects were reared on potato plants (*Solanum tuberosum* var. Kennebec) at 25 °C, 50% relative humidity under a 16L:8D photoperiod. All bioassays were conducted under these same conditions. Adult beetles were selected for the study due to their higher levels of insecticide tolerance compared to larvae [39] and all experiments used individuals that had emerged less than seven days prior.

### 2.2. Chemicals

Noromectin, an injectable 1% ivermectin solution formulated as an antiparasitic for livestock, was obtained from Norbrook (Newry, UK) and was diluted in deionized water for feeding assays. Verapamil was acquired as a ≥99% purity powder from Sigma-Aldrich (St. Louis, MO, USA) and dissolved in 99% ethanol.

### 2.3. Identification of MDR Genes in L. decemlineata

Potential MDR genes in *L. decemlineata* were identified using the BLAST program and QIAGEN CLC Genomics Workbench software by comparing known MDR transcripts from cabbage looper, *Trichoplusia ni*, Asian long-horned beetle, *Anoplophora glabripennis*, leaf beetle, *C. tremula*, and red flour beetle, *Tribolium castaneum*, to two *L. decemlineata* transcriptomes [40,41]. Potential genes identified were then aligned to the transcriptome produced from the *L. decemlineata* genome [38] to assemble the complete ABCB gene set.

### 2.4. Tissue Expression of MDR Genes in L. decemlineata

The expression levels of the genes encoding full MDR transporters were determined in midgut, MT, and nerve tissue to localize where each gene is primarily expressed. Beetles were anesthetized on ice and then dissected in insect saline solution (pH 7.2, 10.7 mM NaCl, 25.8 mM KCL, 90 mM glucose, 29 mM CaCl_2_, 20 mM MgCl_2_, and 5 mM HEPES). The heads were removed to obtain nerve tissue, while midgut and MT tissues were obtained via an anterior-posterior incision along the ventral side of the body. Each replicate of head or midgut tissues included material from three insects, while a replicate of MT tissue included material from twelve insects due to the small volume of tissue. Two or three replicates of each tissue type were used. All tissues were placed in RNAlater solution, stored at 4 °C overnight, and then held at −20 °C for long term storage until total RNA was extracted using the QIAGEN RNeasy mini kit. This RNA was used for cDNA synthesis and qRT-PCR to measure the relative expression of each gene in each tissue (Table S1).

### 2.5. Double-Stranded RNA Synthesis

Double-stranded RNA was produced in vivo through bacterial expression using *Escherichia coli* HT115(DE3) cells transformed with the RNAi vector plasmid L4440 (Addgene plasmid #1654) [42]. The vector was a gift from Andrew Fire (Stanford University). Pairs of primers (Table 1) for PCR amplification of a 330 bp fragment of *LedMDR1*, a 393 bp fragment of *LedMDR2*, and a 411 bp fragment of *LedMDR3* were designed using sequences from the ABCB gene set. The fragments were chosen from areas that had low homology among the three transcripts to minimize possible cross-target effects on different MDR transcripts. The primers included recognition sites for *Not* I and *Sal* I restriction enzymes on the forward and reverse primers, respectively, to facilitate insertion into the L4440 plasmid. Each fragment was amplified by PCR using a mixture of head, midgut, and MT cDNA as template before being cloned with the L4440 plasmid and *E. coli* HT115(DE3) cells using standard methods. *E. coli* HT115(DE3) cells transformed with a L4440 plasmid containing an insert of a 449 bp fragment of the green fluorescent protein (GFP) gene were used as a control for nonspecific dsRNA effects.

Cultures of the transformed *E. coli* strains were induced using isopropyl β-d-1-thiogalactopyranoside (IPTG) to promote dsRNA synthesis. For each strain, 20 mL of an overnight culture of the transformed bacteria were inoculated into 1 L of LB + 100 μg/mL ampicillin + 12.5 μg/mL tetracycline, and 400 μL of the overnight culture were also inoculated into 20 mL of the same medium to serve as an uninduced control. Both cultures were incubated at 37 °C while shaking at 180 rpm. One mL of 1 M IPTG was added to the 1 L culture at an OD600 of 0.5, and incubation of both cultures with shaking was continued for 4 h. Cells from 500 μL samples of both cultures were collected by centrifugation and total RNA extracted using the Epicentre MasterPure Complete DNA and RNA Purification Kit. The RNA was visualized by electrophoresis on a 1.2% agarose gel comparing induced to noninduced samples to verify that the dsRNA fragments were successfully synthesized (Figure S1). The remainder of the 1 L induced culture was centrifuged at 10,400 g, 10 °C to pellet the cells. The pellets were washed with phosphate buffered saline (PBS), resuspended in 100 mL of PBS, divided into 10 mL aliquots, and then stored at −80 °C until being used for feeding assays.

### 2.6. Feeding and Survival Assays

#### 2.6.1. RNAi Silencing Assays

Insects were fed untreated potato foliage, foliage treated with non-dsRNA-expressing HT115(DE3) bacteria, or foliage treated with bacteria producing one of four dsRNA fragments: *GFP* dsRNA, *LedMDR1* dsRNA, *LedMDR2* dsRNA; or *LedMDR3* dsRNA. Foliage was treated by briefly submerging leaf clippings in the appropriate bacterial cultures, then allowing them to air dry. Foliage for each treatment was then placed in separate 150 mm Petri dishes with air holes in the lid and moist filter paper at the bottom. Four or five beetles per replicate were placed in each dish with the foliage and allowed to feed ad libitum. Six dishes (replicates) were used per treatment type. The food was replaced every 24 h for 72 h, at which point three beetles from each dish were removed and dissected for head, midgut, and MT tissues. All tissues from the three insects were pooled together for RNA extraction and qRT-PCR (Table S2). Thus, pooled tissues from three insects made up each of the six replicates.

#### 2.6.2. dsRNA + Ivermectin Survival Assays

To test for ivermectin toxicity after gene silencing, insects were fed untreated potato foliage or foliage treated with bacteria expressing one of the four previously described dsRNA fragments. A total of 24 to 26 beetles for each treatment were fed for 72 h using the same method as the RNAi silencing assays. After feeding on the dsRNA, the beetles were separated into individual 100 mm Petri dishes with moistened filter paper and were provided with an 8 mm diameter leaf disc that had been treated with 1 µL of 20 ppm (20 ng) ivermectin. The dose was chosen to yield an intermediate level of mortality based on preliminary toxicity screening performed on adult *L. decemlineata* (Table S3). The insects were allowed to feed on the disc overnight to ensure every insect completely consumed the entire dose of insecticide, after which they were fed untreated potato leaf clippings ad libitum. Insects were observed daily for seven days for intoxication (unable to walk forward a body length) or death. Intoxicated beetles that did not recover within seven days were deemed moribund and counted as dead when analyzing the data. Each beetle was considered a replicate. Kaplan–Meier estimators were used to model the survival of the insects in each treatment and a log-rank test was performed to compare the survival rates.

#### 2.6.3. Verapamil + Ivermectin Survival Assays

Verapamil was used to inhibit the activity of ABC transporters at the protein level to verify if phenotypic changes caused by direct transporter inhibition would be similar to those caused by transcript downregulation. Verapamil is a common pharmacological inhibitor of ABC transporters used to test ABC transporter activity in a variety of organisms, including insects [17,18,19,20,21,23,24,26,27,28,29,30]. In most species, verapamil produces a synergistic effect with insecticide in both resistant and sensitive populations. Of approximately 350 beetles, verapamil was administered to two thirds of the individuals by feeding a leaf disc pretreated with 100 ng of verapamil (inhibitor was applied in ethanol and allowed to dry) before insecticide exposure. This was the maximum quantity of verapamil adult insects would readily consume, and represents a stoichiometric five to one excess over the ivermectin dose. The remaining third of the beetles were fed a leaf disc treated with ethanol only. The insects were allowed to feed overnight and the next morning half of the verapamil treated beetles were again fed a leaf disc pretreated with verapamil only, and the other half were fed a disc pretreated with both verapamil and 1 µL of 20 ppm ivermectin. All of the beetles previously fed vehicle only (ethanol), were then fed a disc pretreated with 1 µL of 20 ppm ivermectin and no verapamil. The resulting three treatment groups were: Verapamil only, ivermectin only, and verapamil plus ivermectin. All beetles were then fed potato leaf-clippings ad libitum and observed for seven days for intoxication and death, with statistical analysis being performed using Kaplan–Meier estimators as described for dsRNA treatments.

### 2.7. cDNA Synthesis

Total RNA samples were DNase-digested using the Ambion Turbo DNA-free kit prior to cDNA synthesis in order to remove any contaminating DNA. cDNA was then synthesized from 1 µg of RNA per 20 uL reaction volume using Invitrogen SuperScript III First-Strand Synthesis SuperMix for qRT-PCR following the manufacturer’s instructions.

### 2.8. qRT-PCR

The Bio-Rad SsoFast EvaGreen Supermix kit was used for all qPCR experiments. Primer pairs for each gene of interest were designed such that each amplicon was located outside of the sequences used for the dsRNA fragments to avoid false positives. The L8e ribosomal subunit gene was used as a reference gene [40]. Amplification efficiency of primers was determined using previously described methods [43]. qPCR reactions were performed using 500 nM primer concentration and 2.5 µL of 1:2 diluted cDNA in 10 µL reactions. The following thermal profile was used: An initial step of 95 °C for 3 min, then 40 cycles of 95 °C for 10 s, 60 °C for 30 s, and 72 °C for 30 s. A melt curve analysis was performed from 65–95 °C to ensure that only one product was present. No-template controls were included for every primer pair. Each reaction was performed in triplicate. The ΔΔCq method was used to analyze the expression data [44]. When measuring localized expression of MDR transcripts in *L. decemlineata*, ΔΔCq values were calculated relative to the tissue with the highest expression for each gene. When measuring expression of genes after RNAi silencing, ΔΔCq values were calculated relative to the expression in insects fed untreated potato leaves. To determine statistical significance, one-way ANOVA and Tukey’s HSD tests (*p* < 0.05) were used to determine statistically significant differences between ΔCq values.

## 3. Results

### 3.1. MDR Genes Identified in L. decemlineata

Potential MDR genes were initially identified in *L. decemlineata* by comparison of transcript data to known MDR transcripts from other insect species, and then reconciled with the complete genome [38]. MDR genes belong to the B subfamily of ABC transporters and seven members of this family were found to be present in *L. decemlineata* (Table 2), compared to six identified in the model coleopteran, *Tribolium castaneum* [45]. Four of the seven genes identified represent mitochondrial type half-transporters, consisting of a single transmembrane domain and ATP-binding cassette, which are thought to be involved in iron homeostasis and protection against oxidative stress [25]. This matches the complement of these genes in *T. castaneum* [45]. The three remaining genes represent full MDR transporters (also known as P-glycoproteins, or P-gps [46]), consisting of two transmembrane domains and ATP-binding cassettes in each, which, based on functions in other species, would be likely candidates to provide efflux activity for defense against xenobiotics [47]. The genes were named *LedMDR1*, *LedMDR2*, and *LedMDR3* (GenBank accessions BK010703, BK010704, and BK010705). Amino acid sequences translated from these transcripts showed the conserved domains typical of MDR proteins: ABC transmembrane domains, ATP-binding cassettes, Walker A and B motifs; and ABC transporter signature motifs.

### 3.2. LedMDR Genes Show Variable Tissue Expression

**Figure 1:**
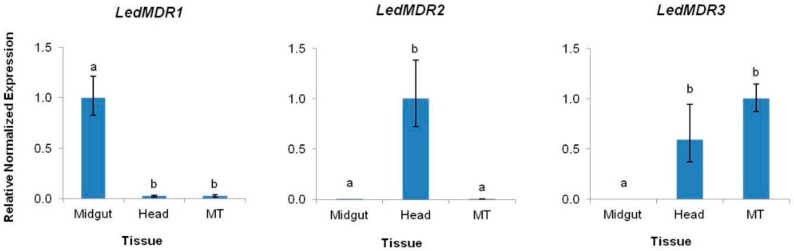
Expression levels of *LedMDR1*, *LedMDR2*, and *LedMDR3* in the midgut, head, and Malpighian tubules (MT) tissues of adult *L. decemlineata*. Bars represent the mean normalized fold change of each gene (± SEM, *n* = 3) relative to the tissue in which each gene is most highly expressed. Different letters represent significantly different expression levels (Tukey’s Honestly Significant Difference (HSD), *p* < 0.05).

To characterize the expression profiles of each of the three new full transporter LedMDR genes in *L. decemlineata*, qPCR was used to measure their relative expression levels in three tissues: Midgut, head, and MT. *LedMDR1* had significantly higher expression (approximately 40-fold) in midgut tissue than the head and MT (F_(2,6)_ = 87.3, *p* < 0.0001), while LedMDR2 had significantly higher expression (300–500 fold) in head tissue when compared to midgut and MT tissues (F_(2,6)_ = 25.5, *p* < 0.01) (Figure 1). *LedMDR3* had similar levels of expression in the head and MT that were significantly higher than in the midgut, where there was no detectable expression (F_(2,5)_ = 1760, *p* < 0.0001) (Figure 1).

### 3.3. LedMDR Genes Were Effectively Silenced by Ingested dsRNA

**Figure 2:**
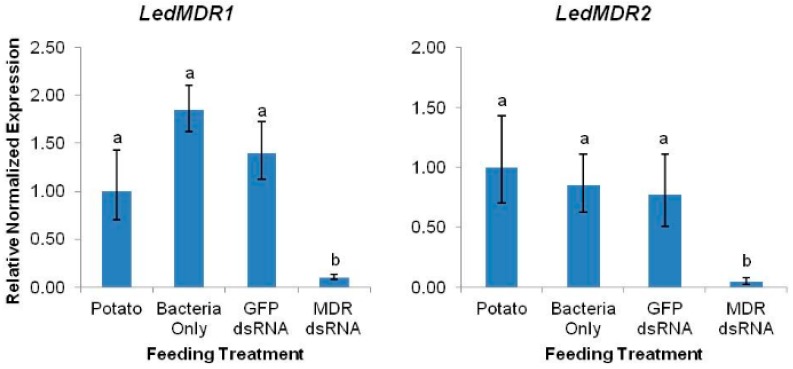
Expression levels of *LedMDR1* and *LedMDR2* in adult *L. decemlineata* fed with dsRNA. Expression was measured in combined midgut, head, and MT tissues after three days of feeding on the indicated treatment. Bars represent the mean normalized fold expression (±SEM, *n* = 6) relative to expression in insects fed with potato. Different letters represent significantly different expression levels within each gene (Tukey’s HSD, *p* < 0.05).

RNAi through feeding of bacterially-expressed dsRNA fragments was used for downregulation of *LedMDR1*, *LedMDR2*, and *LedMDR3* in adult beetles. The insects were fed one of the following treatments: Untreated potato foliage, potato foliage treated with non-dsRNA-expressing bacteria, potato foliage treated with GFP dsRNA-expressing bacteria, or potato foliage treated with bacteria expressing dsRNA specific to *LedMDR1*, *LedMDR2*, or *LedMDR3*. The relative expression levels of each gene in the combined midgut, head, and MT tissues of insects were measured using qPCR after each feeding treatment. *LedMDR1* and *LedMDR2* were significantly downregulated in beetles after ingestion of bacteria containing respective gene-specific dsRNA fragments compared to those fed with any of the other treatments (*LedMDR1*: F_(3,20)_ = 32.7, *p* < 0.0001; *LedMDR2*: F_(3,20)_ = 69.5, *p* < 0.0001) (Figure 2). No significant differences were observed between control treatments. Compared with potato-fed controls, *LedMDR1* and *LedMDR2* expression was 90% and 96% silenced, respectively. *LedMDR3* expression could not be measured effectively in the silencing assays as the transcript abundance was too low for qPCR to provide consistently accurate measurements in the samples combining RNA from all three tissues.

### 3.4. Neither Silencing of MDR Genes nor MDR Protein Inhibition Increased Toxicity of Ivermectin in L. decemlineata

**Figure 3:**
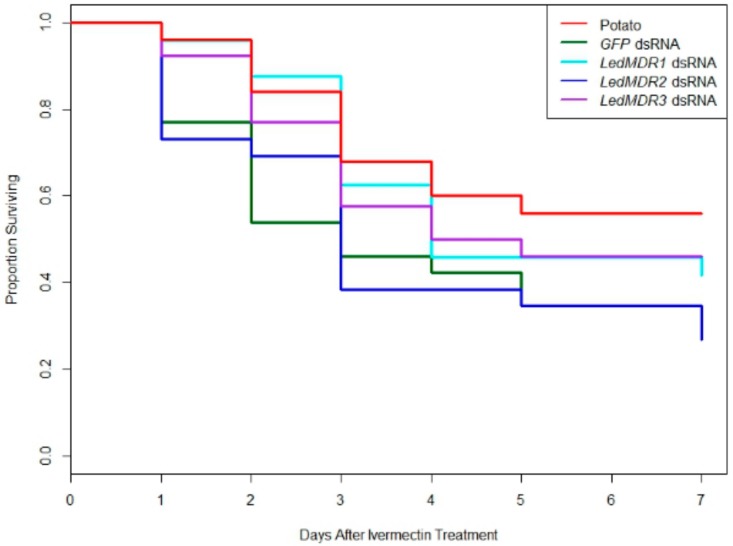
Proportion of adult *L. decemlineata* surviving in different dsRNA feeding treatments each day after consuming ivermectin. *L. decemlineata* adults were fed with the indicated treatment for three days before being given a 1 µL dose of 20 ppm ivermectin. No significant differences were observed between treatments (log-rank test, χ^2^(4, 24) = 7.5, *p* = 0.1).

**Figure 4:**
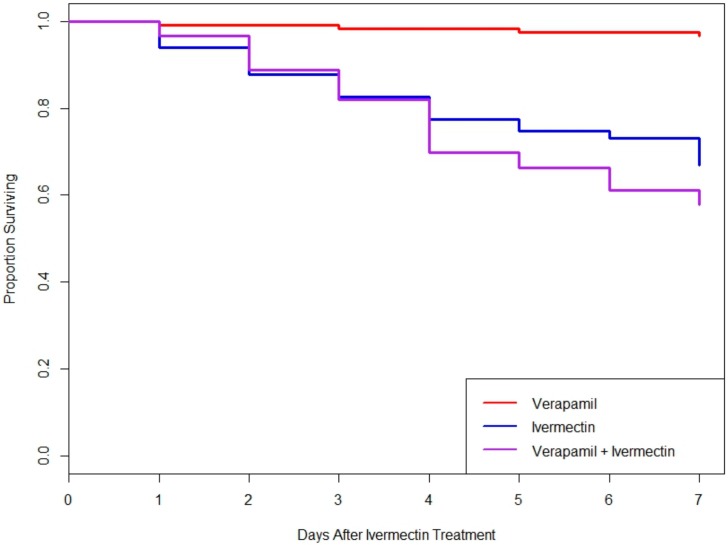
Proportion of adult *L. decemlineata* surviving in different verapamil and ivermectin feeding treatments. No significant differences were observed between the ivermectin and verapamil + ivermectin groups (log-rank test, χ^2^(1, 115) = 1.8, *p* = 0.2).

To test if downregulation of LedMDR genes or inhibition of LedMDR proteins had an effect on toxicity for the insecticide ivermectin in *L. decemlineata*, beetles were fed a dose of ivermectin after being fed one of the dsRNA feeding treatments or a dose of verapamil. When fed on dsRNA, no significant differences in survival patterns were observed between any of the groups (log-rank test, χ^2^(4, 24) = 7.5, *p* = 0.1) (Figure 3). Similarly, receiving verapamil prior to and during a challenge with ivermectin did not significantly change survival when compared to those treated with ivermectin without verapamil (log-rank test, χ^2^(1, 115) = 1.8, *p* = 0.2) (Figure 4).

## 4. Discussion

In this study, seven ABCB subfamily transporter genes were identified in *L. decemlineata* using transcriptomic data in combination with published genome data. Although only a few species of insects have been comprehensively investigated for all ABCB genes, 4–9 ABCB genes have been identified in other insect species [25], including six in the model coleopteran *T. castaneum* [45]. Comparing the two beetle species, three efflux transporter genes of the full two-module type were found in *L. decemlineata* compared to two that are present in *T. castaneum*. Using scaffold data from the CPB genome, *LedMDR2* and *LedMDR3* were found to be adjacent to each other in the genome, suggesting these two genes represent a recent duplication, expanding the ABCB family in this species relative to *T. castaneum*. The varied patterns of expression of the MDR genes we observed between different tissues in *L. decemlineata* are typical of results reported in other species. In *Chrysomela populi*, another leaf beetle, the transcript abundance of 65 potential ABC transporter genes has been examined between gut, MT, fat body, and glandular tissue [48]. Most of the genes in *C. populi* varied in expression by tissue type with some having markedly higher expression in a single tissue and others being more evenly expressed throughout all the tissues. Similar results were found in *Anopheles gambiae*, in which ABC transporter genes had unique tissue expression profiles between abdomen, midgut, MT, and other tissues. Notably, ABCB transporters had higher expression in midgut and MT tissues compared to the rest of the body [49].

The specific expression profile of each MDR gene can be indicative of the function and possible substrates for its corresponding protein. *LedMDR1* was primarily expressed in midgut tissue, so it may serve to protect against ingested compounds which could include plant secondary metabolites (PSMs). *Heliothis virescens* and *T. ni* excrete nicotine or its metabolites when feeding on tobacco plants [50,51], *Lymantria* sp. excrete and sequester camptothecin when feeding on *Nothapodytes nimmoniana* [52], the tortoise beetle, *Eurypedus nigrosignata*, excretes secondary metabolites from its host plant into fecal shields for protection [53], and an ABC transporter in *C. populi* was specifically identified as necessary for sequestration of plant-derived metabolites used in defense [54]. These examples illustrate how insects can manage PSMs by excretion or sequestration, which can be accomplished by ABC transporters such as MDRs. LedMDR1 could potentially serve such a role in *L. decemlineata* based on its localization to midgut tissue.

LedMDR2 could protect against neurotoxins as its gene was primarily expressed in the head, an area containing concentrated nervous tissue. ABC transporters often serve to prevent nerve toxins from entering the brain or CNS. MDR genes have been identified in *Drosophila melanogaster* [55] and *Manduca sexta* [56], where they are expressed in the CNS-humoral interface, the blood-brain barrier equivalent in insects. Reduced penetration of nerve-targeting xenobiotics such as nicotine was correlated with expression of these genes in both insects. Mammalian MDRs have also been shown to protect against nerve toxins in dogs [36], mice [57,58], rats [15], and bovine brain capillary endothelial cells [59]. LedMDR2 may serve a similar function in *L. decemlineata*.

LedMDR3 could have a more general detoxification role, as its gene was expressed in both the head and MT tissue. Expression of MDR genes in the MTs of two flies, *D. melanogaster* and *Mayetiola destructor*, has been linked to detoxification of PSMs [60,61]. Excretory mechanisms in the MT tissues also protect against nicotine and vinblastine in *M. sexta* [62] and nicotine in the hemipteran *Rhodnius prolixus* [63]. Verapamil, an ABC transporter inhibitor, reduces excretion in both species, implying that MDRs could be contributing to this protective activity. In the diamond back moth, *Plutella xylostella*, a MDR homolog was also linked to macrocyclic lactone resistance [64]. Since the MTs act as the primary filtration and excretory tissue for insects, LedMDR3 may eliminate xenobiotics that are not substrates of more specific transporters and could also have a redundant function to eliminate ingested xenobiotics that gut transporters fail to excrete.

Precedents exist for MDR genes expressed in gut, nerve, and MT tissues to be involved in xenobiotic detoxification, so all three *L. decemlineata* MDR genes identified here are possible contributors to insecticide defense. Even so, the fact that toxicity of ivermectin was not increased due to RNAi silencing or verapamil inhibition implies that these transporters do not play a significant role in ivermectin detoxification in *L. decemlineata*. In contrast, MDR gene expression and protein activity have been identified as important factors in detoxification of ivermectin and other macrocyclic lactones in a multitude of other species, including dogs [36] and mice [57,58], where MDRs help prevent ivermectin penetration through the blood-brain barrier and into nerve tissue. MDR expression was also linked to ivermectin resistance in the nematodes *Caenorhabditis elegans* [65] and *Haemonchus contortus* [66], and in many arthropods, including the tick *Rhipicephalus (Boophilus) microplus* [30] and the model insect *D. melanogaster* [29], while in the agricultural pests *H. armigera* [67] and *Spodoptera exigua* [68], either gene expression or protein activity has been linked to ivermectin or abamectin resistance. In *L. decemlineata*, genome analysis has shown there is significant expansion of the ABCC subfamily of ABC proteins [38], making these multidrug resistance-associated proteins (MRPs) also candidates for providing defense against an insecticide such as ivermectin. However, efflux transporters of this subfamily should also be inhibited by the blocker verapamil, so any effects of those transporters would be limited at best since the use of verapamil did not produce a statistically significant effect on ivermectin toxicity in our experiments.

Several other enzyme families exist that could potentially be relevant for tolerance to ivermectin in *L. decemlineata* and genes that contribute to tolerance for other macrocyclic lactones such as avermectin or abamectin are likely candidates. CYP gene expression and enzyme activity have been implicated in avermectin resistance in *L. decemlineata* [69], the silkworm, *Bombyx mori* [70], and the diamondback moth, *P. xylostella* [71]. Increased activity of GST enzymes has been connected to avermectin resistance in the scabies mite, *Sarcoptes scabiei* var. *hominis* [72], and the vegetable leafminer, *Liriomyza sativae* [73]. In *T. urticae* and *R. microplus*, CYP, GST, and esterase enzymes were all shown to be linked to abamectin or ivermectin resistance [28,33,74,75]. Clearly, macrocyclic lactone detoxification can involve different enzymes depending upon the species and in many instances, overexpression of these genes has been linked to resistance to other insecticides as well. For example, CYP, UGT, and GST have been directly linked to imidacloprid resistance in *L. decemlineata* [40,76,77]. However, in our studies of *L. decemlineata* populations showing field evolved resistance to neonicotinoids, we found no evidence of upregulation of MDR transporters in the resistant population, but we did find upregulation of a member of the G-subfamily of ABC transporters. Similarly, Gaddelapati et al. [78] found members of the ABCG and ABCH subfamilies upregulated in a similar neonicotinoid resistant population when compared to a sensitive population. In examining temporal seasonal stages of imidacloprid resistant *L. decemlineata* in Wisconsin populations of these insects, Clements et al. [79] also identified an ABCG transcript whose upregulation was associated with resistance. In combination with the results presented here on innate insecticide defense against a macrocyclic lactone, the evidence suggests that *L. decemlineata* does not typically make use of MDR or MRP family transporters for insecticide defense. However, in contrast to these results, a recent study found both ABCB and ABCC transcripts to be upregulated in two imidacloprid resistant *L. decemlineata* populations from the Central Sands region of Wisconsin [80]. This suggests that these phase III excretion systems can in fact potentially be upregulated to support the action of phase I and II detoxification systems in populations chronically exposed to insecticides.

## 5. Conclusions

This research identified genes encoding three full MDR transporters in *L. decemlineata* that each had unique tissue expression profiles, suggesting different functions. Their potential involvement in ivermectin detoxification was investigated through RNAi gene silencing and pharmacological protein inhibition, but no correlation was found. Although ivermectin toxicity did not change as a consequence of MDR inhibition, detoxification also involves other enzymes such as CYPs, UGTs, and GSTs, which often act together with efflux transporters such as MDRs. Investigating them together, instead of in isolation, might provide more insight into ivermectin detoxification in *L. decemlineata*. Furthermore, the roles of other phase III transporters of the ABCG and H families could be examined as these molecular subfamilies have been shown to be more frequently involved with resistance to neonicotinoids such as imidacloprid. Knowledge of how phase III excretion systems can be amplified to support insecticide metabolism will be essential to developing strategies to mitigate resistance development.

## Figures and Tables

**Table 1:** Primers used for double-stranded RNA (dsRNA) fragment synthesis and qPCR expression measurement of *L. decemlineata* ABCB (LedMDR) genes in different tissues of the Colorado potato beetle.

Primer	Sequence
Primers for Fragment + L4440 Construction	
*LedMDR1* For	[GAGCGGCCGC]TGTTCATGATTTATTCTAGT *
*LedMDR1* Rev	[GCAGGTCGAC]ATCTGGTCTGACGGATAG *
*LedMDR2* For	[GAGCGGCCGC]AAGTTAGCCGTGGAAGCCAT *
*LedMDR2* Rev	[GCAGGTCGAC]TCCCACGCACAATTCACTGG *
*LedMDR3* For	[TAGCGGCCGC]AGTGGGAAGACGCCATCAGT *
*LedMDR3* Rev	[GCAGGTCGAC]TGCCATACCACCAACATAACGA *
qPCR Primers	
q*LedL8e* For	GGTAACCATCAACACATTGG
q*LedL8e* Rev	TCTTGGCATCCACTTTACC
q*LedMDR1* For	TAGACCTCACATGGTTCAGG
q*LedMDR1* Rev	TTAGACTTCCGTTGACTTCTTC
q*LedMDR2* For	TAGTTTCCCAGGAGCCGAAC
q*LedMDR2* Rev	TTCGCACTCTTTGCAGCTTTC
q*LedMDR3* For	TCGTTGGTATCTGCTCTCTTCG
q*LedMDR3* Rev	TGAGGTGCCATTATTCGATCTG

* Sequences added for restriction enzyme cloning are indicated by square brackets.

**Table 2:** Potential MDR genes (ABCB subfamily) identified in the *L. decemlineata* genome.

Name	Protein Type	Accession	Genome Transcript(S) *
*LedABCB 1*	Full	BK010703	LDEC021233/014301
*LedABCB 2*	Full	BK010704	LDEC008907/008908/008909/008910
*LedABCB 3*	Full	BK010705	LDEC008906
*LedABCB member 6, mitochondrial-like*	half	XM_023161180	LDEC007355/022912
*LedABCB member 7, mitochondrial-like*	half	XM_023169211	LDEC002144/024539
*LedABCB member 8, mitochondrial-like*	half	XM_023160888	LDEC019485
*LedABCB member 10, mitochondrial-like*	half	XM_023173355	LDEC024415/010427

* from Schoville et al. [38].

## Supplementary Materials

The following are available online at https://www.mdpi.com/2075-4450/11/2/135/s1. Figure S1: Comparison of extracted total RNA from induced and noninduced bacterial cultures verifying success of dsRNA fragment synthesis. Table S1: Raw qRT-PCR data for measurement of relative expression levels of each MDR gene in each tissue tested. Table S2: Raw qRT-PCR data for measurement of relative expression levels of MDR genes in silencing experiments. Table S3: Raw survival data for an ivermectin toxicity screen performed on adult *L. decemlineata*.
