# Supplementary figures and images for: The ABCB Multidrug Resistance Proteins Do Not Contribute to Ivermectin Detoxification in the Colorado Potato Beetle, Leptinotarsa decemlineata (Say)
Source: Insects. 2020 Feb 20;11(2):135. doi: 10.3390/insects11020135 (PMC7074147; doi:10.3390/insects11020135)

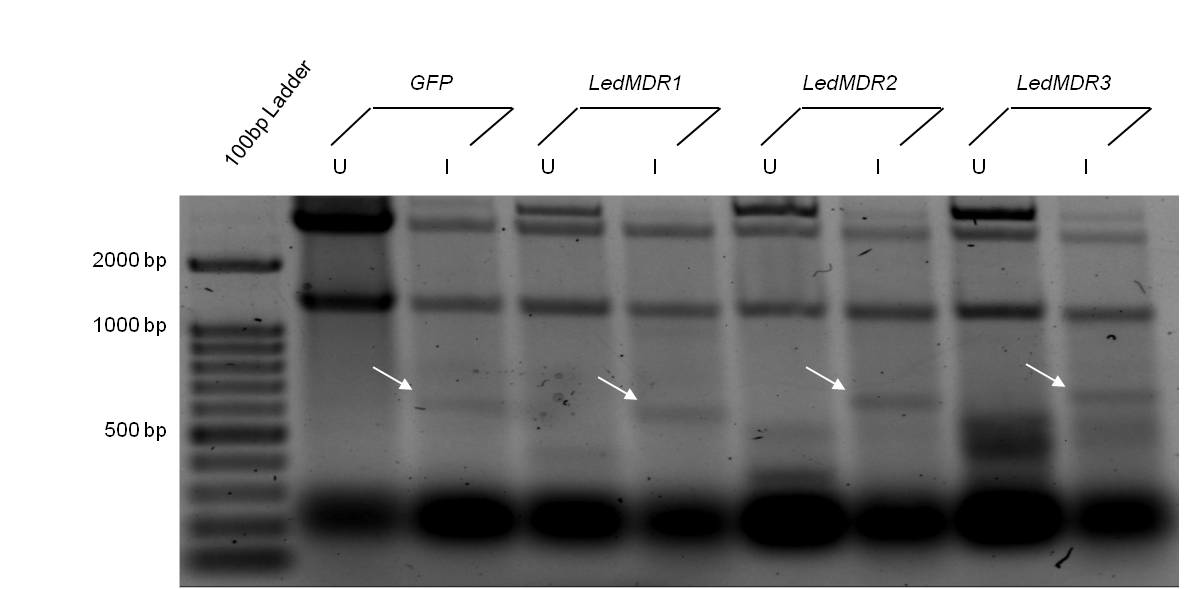

Supplement: Supplementary file 1 [file insects-11-00135-s001.zip › Figure S1.tif]
